# Can digital finance boost SME innovation by easing financing constraints?: Evidence from Chinese GEM-listed companies

**DOI:** 10.1371/journal.pone.0264647

**Published:** 2022-03-03

**Authors:** Lianying Yao, Xiaoli Yang

**Affiliations:** 1 School of Economics, Zhejiang University of Technology, Hangzhou, China; 2 Global Institute for Zhejiang Merchants Development, Zhejiang University of Technology, Hangzhou, China; 3 School of Economics, Wuhan University, Wuhan, China; DePaul University, UNITED STATES

## Abstract

This paper summarizes the transmission chain of “digital finance-financing constraint-firm innovation” at the theoretical and practical levels, incorporates digital finance into the empirical analysis framework of firm innovation, selects the data of Chinese GEM(Growth Enterprise Market)-listed companies from 2011 to 2020, and matches the data of the digital inclusive finance index. The paper empirically examines the incentive effect and impact mechanism of digital finance on SME innovation through the two-way fixed-effects model and mediated-effects model by matching the data of China GEM-listed companies from 2011 to 2020 with the digital financial inclusion index data. The findings show that the digital development and promotion of digital finance play a significantly positive impact in helping SMEs innovate and stimulate innovation. The effect is realized by alleviating corporate financing constraints. Further, digital finance has different incentive effects on enterprises with varying rights of property nature, as well as on other regions.

## I. Introduction

Chinese small and medium-sized enterprises (SMEs) are generally plagued by difficulties with financing. The root of the problem lies in natural deficiencies of China’s current traditional financial system in serving SMEs or private enterprises [1, 2]. Corporate innovation activities inherently have the attributes of significant upfront capital investment, a long development period, and unpredictable research and design (R&D) results, which often require extensive and stable cash flow support. In contrast, SMEs have difficulties obtaining external financing from traditional financial institutions due to objective reasons such as a lack of historical valid financial data, collateralizable assets, and implicit government guarantees; thus, financing constraints further limit SMEs’ innovation [3, 4]. On the one hand, the prolonged nature of trade friction between China and the US has led to elevated external risks; on the other hand, with the disappearance of the demographic dividend, the transformation of the domestic economy from labor factor-driven to innovation-driven has become increasingly urgent. China has proposed a double-loop strategy under the dual influence of internal and external factors. In the face of the transition from the old to the new economy, how can the original financial system better support the real economy and the innovation economy [5, 6]? Digital finance can effectively leverage emerging digital technologies such as big data to analyze and harness the massive amount of user data accumulated in the underlying layer, effectively breaking through the old operational model of China’s traditional financial system, filling in gaps in the conventional financial market to a certain extent, and widening the channels of financing for SMEs [7–9]. Hence, this paper examines whether and how digital finance can alleviate financing constraints and promote innovation among SMEs.

This paper presents an empirical study on the linkages between digital finance, SME innovation, and financing constraints based on the microdata of GEM-listed companies from 2011 to 2020, taking into account the current situation of China’s capital market and social operations, and selecting appropriate financing constraint indicators and related influencing factors. Specifically, the study focuses on the following two questions: First, can digital finance alleviate financing constraints and promote corporate innovation? Second, is the mechanism heterogeneous for firms with different geographic distributions and property rights?

The paper’s theoretical contribution is that research on the impact of financing constraints on corporate innovation is more common in academia. However, exploring the relationship between these three components from the perspective of digital finance is still in its infancy. Therefore, based on the existing literature, this paper introduces the financing constraint as a mediating variable from digital finance to broaden the research perspective on the influencing factors of corporate innovation, and to enrich the literature on digital finance and corporate innovation. The paper’s practical significance is that the Chinese economy is currently in a critical development stage of transitioning from a factor-driven model to an innovation-driven model. To effectively play the role of technological innovation in helping China’s economy to become healthy and high-quality under the “new normal,” and supporting the role of digital financial services in the real economy, it is necessary to clarify the relationship between SME innovation and digital finance development and the influencing mechanism. Second, the paper examines how new digital technologies can be used to empower inclusive finance, reach out to the vast “long tail” group, effectively solve the financing constraints faced by SMEs, and find a new way to build traditional inclusive finance. Finally, regulators offer theoretical support to improve the efficiency of regulatory tools and use the “regulatory sandbox” as an effective means to balance financial innovation and financial stability.

This paper explores the differences in the impact of digital finance on the innovation of enterprises with different characteristics from the perspective of the nature of property rights and geographic distribution, and provides empirical evidence for the development of digital finance in China to alleviate the financing constraints of SMEs and promote enterprise innovation.

The innovations of this paper are as follows: First, the research horizon is innovative. Most studies have focused on the impact of traditional finance on enterprise innovation, but there is a lack of research on digital finance and the real economy. This paper takes digital finance as an entry point for research and incorporates it into the framework of the analysis of factors influencing SME innovation, thus enriching the study of SME innovation at the macro level and shedding light on how the growth of financial markets can help promote enterprise innovation. As China’s economy is now at a critical stage in its transition from a factor-driven to an innovation-driven growth model, it is essential to clarify the relationship between SME innovation and the development of digital finance and the mechanisms that influence it. The aim is to effectively leverage technological innovation to support the healthy, high-quality expansion of China’s economy under the “new normal” and to facilitate the role of digital financial services in the real economy.

In terms of theoretical innovation, previous studies on digital finance’s impact on enterprise innovation are primarily direct, and the indirect effect between the two is still in the exploration stage. This paper will clarify the transmission mechanism of “digital finance—financing constraints—enterprise innovation” and how digital finance can alleviate financing constraints and stimulate enterprise innovation. There are many studies on the impact of financing constraints on corporate innovation.

Regarding data innovation, the availability of quantitative indicators limits the existing literature on digital finance, and the data sample is limited to the period from 2011 to 2015. Nevertheless, with the release of the second Digital Finance Index, the period of this paper can be extended to 2020, which expands existing research.

## II. Literature review

### 1. The impact of digital finance on the real economy

Digital finance has played an essential role in improving financial services inclusion [5, 10]. The difficulty and pain point of inclusive finance is how to provide essential financial services to the long-tail customer group of small enterprises and rural grassroots organizations, which have been excluded from traditional financial aid for a long time due to difficulties in credit approval and in controlling loan default risks due to imperfect historical financial information and no pledged assets [11, 12]. The popularity and improvement of digital infrastructure (via the mobile internet and online payments) have created objective conditions for inclusive finance to break through the services dilemma [13]. The new generation of digital technology has a series of natural advantages in solving the barriers to developing inclusive finance. On the one hand, it can transfer the target customer group from offline to online with the help of major internet platforms and use. On the other hand, it can move the target customer group from offline to online with the help of major internet platforms and reduce the cost with its higher user stickiness. Further, it can mine and analyze the massive amount of underlying, non-standard data through digital technology, draw a particular customer portrait, and use various types of “soft information” for credit approval, thus reducing information asymmetry. Compared with traditional financial institutions, online banks’ credit scoring models based on “big data” have more information advantages in credit assessment [14, 15]. [16] argued that digital technology promotes financial inclusion through three main channels: reducing service costs, improving the effectiveness of risk control, and reforming financial services. The “contactless lending service” launched by domestic central banks and financial institutions in response to the epidemic is a typical application of digital financial services; this has significantly improved the coverage and accessibility of financial services, erased the time and spatial constraints between financial institutions and customer groups, and involved the use of modern digital technology to bring services to those were initially deprived of financial services. Modern digital technologies have brought long-tail customer groups, which have been financially excluded, within the reach of formal financial institutions [16, 17]. Taking advantage of the “wind” of the epidemic, an increasing number of offline banking services are being replaced by “contactless financial services” offered by online banking [18]. [19] asserted that via “long tail theory” analysis, digital finance mainly solves the financing problem of SMEs through two paths: complementing the shortage of traditional financial services and optimizing resource allocation. In addition, [20] pointed out that digital currency, as a form of digital finance development, can effectively improve the coverage of financial products and help financial services to reduce costs and increase efficiency.

At present, digital finance development models and business forms show a diversified trend and have achieved a high degree of penetration into our daily lives [21]; while digital finance is a central hot research area, its related theories and practices have received much scholarly attention [22]. [23] found that the growth of digital finance is beneficial to the expansion of the real economy, and the impact path is shown as a cubic curve. Moreover, the impact has spatial spillover effects. Subsequently, [24] confirmed the above view. Further, they explored the mediating development that R&D innovation plays in spatial spillover effects, and finally, they found structural differences in digital finance in boosting the real economy. Due to its inclusive nature, digital finance contributes to the development of rural finance [25] and plays a significant role in reducing the income gap between urban and rural residents [26, 27], while the gap between urban and rural consumption decreases in parallel with the income gap. The transmission relationship between residential consumption and digital finance may be realized through the two paths of providing more convenient means of payment and easing the liquidity constraint of residents [2]. Importantly, along with the increase in residential consumption, there is accelerated growth in residential household debt, which needs to be guarded against. [28] found that digital finance can effectively curb firms’ inefficient investment behavior, and the specific transmission mechanism is achieved by helping firms reduce their leverage and improve their level of financial stability [29].

### 2. The impact of digital finance on innovation and entrepreneurship

Digital finance can generate economic value by promoting technological innovation and supporting entrepreneurship. Research in this area is still in the exploratory stage, but some interesting findings have been obtained. The lack of support from traditional financial services, including innovation and entrepreneurship, has largely limited social entrepreneurial activities [30]. The recent advances in digital technology and the relatively relaxed regulatory environment in China have led to the flourishing of digital finance as a new financial model [31, 32]. The flourishing of digital finance as a novel economic model has dramatically improved long-standing financial exclusion in China, allowing long-tail customers who were previously excluded from the financial system to enjoy convenient access to essential financial services [33]. To date, research on the factors influencing entrepreneurship is well established, but the analysis of the impact of digital finance on entrepreneurship is still in its infancy [34].

SMEs, as the more active agents of innovation activity, have received extensive scholarly attention. Financial services provide the financial support needed for entrepreneurial activities, while technology drives the transformation and upgrading of business models [35]. Digital finance, as a more profound integration of digital technology and financial services, meets both the needs of entrepreneurship and has a uniquely inherent advantage in promoting entrepreneurship. [36] argued that digital finance can rely on big data technology to mine and analyze a large amount of non-standardized data, establish a new set of wind control systems, and input various types of information from different subjects into the model for accurate credit risk assessment. This new assessment model, combined with the characteristics of big data, can effectively compensate for the lack of information required by small enterprises or individuals for financing approval [37], thus reducing the information asymmetry between banks and business partners, and optimizing the rational allocation of financial resources. New digital economic development models, such as network lending and supply chain finance, have helped small enterprises compensate for the lack of credit information to a certain extent, helping them to solve the entrepreneurial obstacle of financial constraints [30], and have proposed new solutions to eliminate the problem of financing difficulties for SMEs.

[38] demonstrated digital finance’s role in promoting innovation and entrepreneurship based on the number of new business registrations per year. [39] built on this finding by explicitly exploring the transmission mechanisms through which digital finance affects innovation and entrepreneurship, ultimately revealing that the relaxation of financing constraints on firms and the increase in government tax rebates are direct drivers of innovation. This effect is heterogeneous across industries and regions. [40] reached a similar conclusion by arguing that digital finance can help external investors in their investment and financing decisions, making it easier for quality firms to obtain credit support, thus stimulating firm innovation.

## III. Theoretical analysis and research design

### 1. The impact of digital finance on innovation in SMEs

The innovation activities of enterprises are inherently characterized by high upfront investment, a long development period, and unpredictable R&D outcomes, which makes R&D investment activities more problematic in terms of information asymmetry compared to fixed asset investment, which is highly deterministic [41]. On the one hand, the primary input element of R&D and innovation activities is the human capital of a company’s technological developers, which, as an intangible asset, is often difficult to objectively assess and accurately measure in terms of its intrinsic value. Moreover, banks and other financial institutions do not accept human capital, as an intangible asset, as eligible collateral [29]. On the other hand, the process of R&D and innovation is a trade secret for a company’s operations and long-term growth. Companies prefer to keep the use of funds and the progress of technological development confidential. Even if companies disclose information, the details are scarce, which results in high regulatory costs for external investors, further exacerbating the already existing information asymmetry and ultimately making it more difficult for companies to raise funds from external financial institutions [42]. It is now widely accepted in industry and academia that financing constraints significantly reduce firms’ ability to innovate and that a well-developed, multilevel financial system will help to alleviate financing constraints on firms, as well as to lift them out of financial distress, thus helping to increase their willingness to innovate [43, 44]. Digital finance, as a new financial industry, effectively fills in the gaps in the traditional financial system, and will hopefully reduce the level of corporate financing constraints and further stimulate corporate innovation in the following ways.

First, the development of digital finance can effectively reduce the information asymmetry between enterprises and financial lenders. The adequacy of a company’s internal capital and the effectiveness of external financial markets determine whether the company can obtain sufficient funds to invest in R&D activities [45]. Due to the information asymmetry that prevails in actual business activities, and the fact that financial markets are not fully efficient, information costs for both the supply and demand of capital arise, thereby limiting firms’ ability to obtain external financing [46]. The prevailing academic view is that information asymmetry is a significant cause of external financing constraints [47]. [48] based on Alibaba’s massive data, found that in addition to traditional credit approval information, Ant Financial captures non-financial soft data, such as consumption information kept by customers on various platforms to assist in getting loans approved. This also effectively reduces the information asymmetry between lenders and borrowers, indirectly serving the real economy and innovative economic growth. Digital finance relies on a new generation of digital technology to quickly capture behavioral information between subjects at different levels, mine useful information, integrate data, and establish an accurate and reliable third-party credit information assessment system [7]. All of the above illustrate the role of digital finance in reducing information asymmetry in market behavior, thus providing external investors with better access to the basis for corporate investment decisions, helping high-quality SMEs obtain credit support, and ultimately promoting corporate innovation and development.

Second, digital finance has broadened access to finance and the financing scale. Digital finance generally refers to the innovative business model of financial services rooted in emerging digital technologies to achieve settlement, investment and financing, and financial management. There are many scattered small-scale market participants in the traditional financial market who comprise the vast long-tail customer groups in the financial market. However, China’s conventional economic system cannot effectively serve these small-scale investors due to cost and technology constraints, resulting in a lack of financial services. Digital finance, with its unique natural advantage of not being restricted by time or space, provides traditional financial institutions with convenient access to such small-scale customers, filling the gap in this area of conventional financial services. Based on the above analysis, digital finance significantly broadens the sources of funds, reduces the degree of credit distortion in the financial market, optimizes the rational allocation of funds, and proposes new solutions to address companies’ financing constraints.

Third, digital finance development can significantly improve the efficiency of credit approval. [49] found that the efficiency of the approval process for rental mortgages in the US increased by almost 20% without increasing the risk of default. [48] based on the massive loan information of Ant Financial, found that Ant Financial, through its pioneering “310” credit approval model, relied on digital finance to significantly reduce the time for financial credit approval and optimize the otherwise cumbersome approval process while also reducing the manual process. This also lowers the cost of manual response and intervention in the business process.

**Based on the above analysis, this paper proposes Hypothesis 1: The development of digital finance can effectively stimulate SME innovation. This stimulating effect is mainly achieved by alleviating SMEs’ financing constraints**.

### 2. The heterogeneity of digital finance’s impact on SME innovation

Under China’s current social system, private enterprises are discriminated against by traditional financial institutions due to their attributes and higher information asymmetry compared to state-owned enterprises, and generally face real problems such as “difficult and expensive financing.” There are significant differences between private and state-owned companies in terms of their strategic level and management model of enterprise growth [50]. In addition to ensuring their expansion, state-owned enterprises often have a social and political responsibility to improve people’s livelihoods and respond to policy calls. At the same time, state-owned enterprises usually have a large scale and a sound internal control system, and their internal financial information is more complete, making it easier to obtain financial support from state-owned banks and the government at low financing costs [51]. On the other hand, due to their property rights, private enterprises face more significant information asymmetries and external borrowing costs than state-owned enterprises due to the higher cost of external regulatory information in the capital market. With the gradual improvement of China’s financial market environment and the establishment of a multilevel capital market, enterprises will face more diverse financing channels. This series of changes will have different impacts on the business and financing decisions of enterprises with varying property rights [52]. Changes in the external financial environment will significantly affect their business strategies, helping them improve the rational use of external funds, optimize the efficient allocation of external financing resources, and further stimulate their innovation and R&D dynamics and efficiency [53]. In the current political, economic, and financial environment, private firms tend to be subject to higher financing constraints than state-owned firms [54].

**Based on the above analysis, this paper proposes Hypothesis 2: Digital financial development, in promoting innovation in SMEs, is heterogeneous across firms with different property rights, and the incentive to innovate is more pronounced for private firms**.

### 3. The geographic distribution of enterprises

There is a clear imbalance in the geographic development of China’s real economy, and finance and the concentration of financial resources, which manifests in the strong east and weak west. At the same time, the financial resources in the Pearl River Delta, the Yangtze River Delta, and the Bohai Sea region are beginning to converge, eventually forming a general trend of radiation development to surrounding cities, with the North, Guangzhou, and Shenzhen as the center. According to previous literature, regional differences in the institutional environment determine, to a certain extent, the degree of financing constraints faced by local firms [55]. In regions with a better institutional environment, a developed financial market environment will significantly reduce the cost of financial services. In contrast, the abundance of financial resources will help them serve as a hotbed for the growth of digital finance, and provide more significant support for new digital technologies. This will help reduce information asymmetries between local SMEs and banks, and increase their likelihood of obtaining external credit support. In contrast, in the central and western regions, where the institutional environment is less favorable, a chronic scarcity of financial resources and financial exclusion is evident. This set of differences in objective conditions will inevitably influence the incentive effect of digital finance on SME innovation in the central and western regions.

**Based on the above analysis, this paper proposes Hypothesis 3: The role of digital finance development, in promoting SME innovation, is heterogeneous for enterprises with different geographic distributions, and the incentive effect on innovation is more evident in the east, where the institutional environment is better**.

### 4. Sample selection and data sources

This paper took GEM-listed companies as the research object for the following reasons: First, the core positioning of GEM is to serve growth-oriented, innovative, and entrepreneurial companies. GEM-listed companies are primarily composed of SMEs. This characteristic is more in line with the paper’s objective. Second, the scale of GEM-listed companies is significant. As of the close of business on November 5, 2021, the number of listed companies reached 916. The distribution is reasonable, including regions with different levels of digital finance development, which is conducive to the analysis of geographic heterogeneity. Third, the availability and authenticity of primarily financial data are considered. Fourth, company patent-related data can be obtained by querying the CNRDS database.

This paper used public data of GEM-listed companies from 2011 to 2020 as the research sample, and other screeners and processes were used to evaluate the original model. First, public utilities, as well as financial listed companies, were excluded due to their unique characteristics in daily operation and asset and liability structure. Second, to eliminate the influence of extreme performance fluctuations on the empirical results, listed companies with ST and ST* in the sample period were excluded. Third, GEM-listed companies with severe deficiencies in relevant financial data were excluded. Fourth, GEM-listed companies with a negative book value of the owner’s equity were excluded. Finally, to eliminate the possible influence of extreme importance, this paper shrunk the tails of the main continuous variables below 1% and above 99% in the original sample. After going through the above steps, 720 listed companies with 3,763 unbalanced panel data observations were finally obtained.

The patent data of GEM-listed companies used in this paper are from the CNRDS database. The digital finance index is from the Digital Finance Research Center of Peking University. The microlevel data of companies are from the Wind database, and the relevant data on provincial characteristics were matched and complemented by the database of Guotaian (CSMAR).

### 5. Variable selection

Explained variable: Firm innovation. The previous research mainly focuses on input and output to measure SMEs’ innovation capability by constructing relevant indicators. More specifically, innovation input can be measured via the R&D investment cost or the number of R&D personnel. Innovation output indicators can be portrayed by the number of patent applications and the output value of new products. To ensure the stability of the empirical findings, this paper’s primary empirical evidence is grounded in the ratio of R&D expenditure to sales revenue (R&D/sales), which is called innovation. The actual technological innovation activities are typically at high risk, and it is not easy to effectively convert R&D inputs into innovation outputs. Thus, the innovation input indicator may be overestimated by the regression results. The number of annual patent applications is a good indicator of the effects of innovation activities. It can better portray firms’ ability and efficiency to convert R&D inputs into results [56]. Based on the above analysis, the reliability of the findings was tested later by replacing the explanatory variables with the innovation output indicator (Patent).

Core explanatory variable: The level of development of digital finance (DIF). The emergence of a new financial industry, digital finance, has improved China’s multilevel capital market and complemented the lack of traditional financial services. The Digital Finance Research Center of Peking University [8], in collaboration with the internet platform Ant Financial Services, has developed a scientific picture of the development level of digital finance in all Chinese provinces, cities, and counties in three dimensions with the help of its massive underlying data, providing a powerful analytical tool for research in digital finance-related fields. Furthermore, it provides a powerful analytical tool for digital finance-related analysis and solves the digital finance measurement problem that has plagued the academic community for many years. In this paper, the provincial-level data of the Digital Finance Index are used as the core explanatory variable.

The mediating variable is financing constraints (SA). The scientific and accurate measurement of corporate financing constraints has long been a significant challenge for academics. [57] was the first to investigate how to quantitatively measure the level of corporate financing constraints, and subsequent research has been conducted in this area.

Cash flow modeling method and the composite index method.

The univariate index method is a simple measure of the level of financing constraints using a single firm characteristic variable. [57] was the first to use investment-cash flow sensitivity as an indicator of financing constraints; this method has been widely discussed by academics for a long time. On this basis, [58] re-examined this indicator construction method and came to a conclusion that was at odds with previous research. They found that firms with fewer financing constraints exhibited greater sensitivity and robust findings.

Firm size has been commonly used to measure financing constraints in earlier studies [59] It is generally believed that external investors prefer to invest in larger firms, which have more access to external finance and suffer from less credit discrimination. [60] used bond credit ratings to portray the level of financing constraints. If a firm can issue bonds in the capital markets and has a high credit rating, it can raise funds at a lower interest rate and has fewer financing constraints. In addition, years of incorporation, the nature of ownership, the dividend payout ratio, the gearing ratio, and interest cover are common proxies used to measure financing constraints [61]. These proxies group a sample of firms into multiple groups, each with different levels of financing constraints. This paper argues that while the grouping of this method is somewhat reasonable and applicable, there are problems with the endogeneity of the selected variables with firms’ level of financing constraints and the relatively one-sided selection of variables.

The main idea of the cash flow model is to construct an econometric model in which the explanatory variable is the company’s cash flow, and the explanatory variable is the company’s cash holding or investment expenditure. While these two models have received empirical support from many scholars, they have also been questioned due to the lack of logical uniqueness and relevance of the cash flow model in determining the level of financing constraints based on the size of the regression coefficients [62, 63].

The central idea is to first classify companies into different groups according to specific quantitative or qualitative indicators, each of which has a different level of financing constraints, and then to conduct a regression analysis on several indicators that can affect the level of financing constraints. Finally, the index is built from the regression coefficients obtained, and the magnitude of the index is used to determine the company’s level of financing constraints. The composite index method has overcome the shortcomings of the cash flow model and the single characteristic variable method to a certain extent. Hence, most scholars currently use this method to measure the level of financing constraints.

[64] considered the limitations of a single indicator and constructed the ZFC index by regressing and weighting six variables using the logistic regression method. Subsequently, [37], based on the work of [58], built the KZ index using ordered logistic regressions on five financial indicators. However, in practice, the KZ index has often been inconsistent with the facts, thereby revealing its shortcomings. As such, [65] constructed the WW index through non-linear GMM estimation. However, the drawback of this index is that it can only gauge equity financing constraints and does not help with bond financing constraints. Since all three indices employ some endogenous variables in their construction and may be biased, [61] constructed a highly exogenous SA index based on an evaluation of previous research results, specifically a combination of firm age and size.

While the composite index has received much support from scholars, many have also questioned the use and validity of some of the indices. Among them, [66] argued that the WW index does not apply to the Chinese capital market and suffers from both endogeneity and a lack of broad applicability. [65] argued that the model estimation of the KZ index is biased and that there are many discrepancies between the descriptive results and objective facts. [67] asserted that the FCP and ASCL indices are not suitable for measuring the level of financing constraints of listed companies, while the SA index is somewhat less challenged. [18] maintained that the SA index can better avoid the endogeneity problem and has better applicability in determining the level of financing constraints of listed companies in China.

To effectively determine the level of financing constraints and ensure robustness, the SA index is used to quantitatively measure the level of financing constraints of non-financial firms in Chinese GEM-listed companies.


$$\mathrm{SA}=0.043{\mathrm{Size}}_{it}^{2}-0.737{\mathrm{Size}}_{it}-0.040{\mathrm{Age}}_{it}$$

In the above equation, Age is the difference between the observation year and the registration year of the company, while Size is the natural logarithm of the company’s total assets. Therefore, the calculation outcome of the SA index is less than 0, and the higher the company’s level of financing constraints, the larger the absolute value of the SA index. In this paper, the total value of the SA index was taken; the larger the final value, the higher the company’s level of financing constraints.

In this paper, the following variables were selected as control variables: return on total assets (ROA); gearing ratio (Lev); firm Size (Size); growth (Growth); fixed asset ratio (PPE); firm’s years of operation (Age); executive shareholding ratio (Share); sole director ratio (Indep); and regional economic development level (PGDP). Table 1 below shows the meaning of each significant variable and the descriptive statistics.

**Table 1:** Definition of primary variables and descriptive statistics.

Variable Name	Variable Symbols	Variable Definition	Average value	Standard deviation	Maximum value	Minimum value
Enterprise Innovation 1	Innovation	R&D expenditure/sales revenue	7.574	6.955	0.57	99.606
Enterprise Innovation 2	Patent	Total number of patent applications	14.988	31.031	0.57	808.22
Digital Finance	DIF	Peking University Digital Inclusive Finance Index	239.284	82.558	33.98	378.954
Return on Total Assets	ROA	Total Profit/Total Assets	7.393	7.101	24.883	24.932
Gearing Ratio	Lev	Total liabilities/total assets	28.903	16.933	4.087	73.829
Enterprise Size	Size	Natural logarithm of the total assets of the enterprise	12.5	1.304	11.12	15.449
Growth	Growth	(Current period net profit—Previous period net profit)/Previous period net profit*100%	4.497	198.247	-1175.43	790.61
Fixed Assets Ratio	PIPE	Total fixed assets/total assets at the end of the year	15.726	11.813	0.922	52.516
Number of years of operation	Age	2020—Year of establishment + 1	15.049	4.871	6.57	27.22
The shareholding ratio of senior management	Share	Number of shares held by executives/total share capital	26.828	20.87	0.57	69585.22
The proportion of sole director	Indep	Number of sole directors/number of board of directors	38.584	5.835	33333.57	58.363
Regional economic development level	PGDP	Natural logarithm of GDP by province	11.708	0.903	10.828	13.071
Financing constraints	SA	SA = -0.737×Size+0.043×Size	-2.693	0.796	-3.239	-1.13

### 6. Model construction

In this paper, when constructing the model for regression, to eliminate the possible influence of uncontrolled industry macro factors on the empirical results, a two-way fixed effects model was selected and applied to the empirical analysis, which fixed the year effect (Year) and industry effect (Industry). Therefore, this paper first analyzed the direct impact of digital finance on corporate innovation, and regression Model (1) was set as follows.


$$\begin{array}{l} {\mathrm{Innovation}}_{i,t}=\alpha_{0}+\alpha_{1}DIF_{m,t}+\alpha_{2}\;{\mathrm{Roa}}_{i,t}+\alpha_{3}\;{\mathrm{Lev}}_{i,t}+\alpha_{4}\;{\mathrm{Size}}_{i,t}+\alpha_{5}\;{\mathrm{Growth}}_{i,t}\\ \;+\alpha_{6}PPE_{i,t}+\alpha_{7}Age_{i,t}+\alpha_{8}J{\mathrm{oint}}_{i,t}+\alpha_{9}M\;{\mathrm{share}}_{i,t}\\ \;+\alpha_{10}\;{\mathrm{Indep}}_{i,t}+\alpha_{11}PGDP_{m,t}+d_{j}+u_{i}+\varepsilon_{i,t} \end{array}$$ (1)

The subscripts i, m, j, and t refer to the firm, province, industry, and year, respectively. The explanatory variable *Innovation_i,t_* is the innovation R&D investment of company i in year t. The explanatory variable *DIF_m,t_* indicates the level of digital finance development in company i’s province m in year t. The other control variables indicate the individual characteristics of company i in year t and the regional-level features of province m. The coefficient *α*_1_ of the core explanatory variable *DIF_m,t_* reflects the overall impact of digital finance development on SMEs’ innovation, which is expected to be significantly positive according to the hypothesis above.

In this paper, financing constraints were incorporated into the framework of mediating effects analysis. A stepwise approach was used to further test and examine the impact mechanism of how digital finance promotes corporate innovation, and regression models (2) and (3) were set as follows.


$$\begin{array}{l} SA_{i,t}=\beta_{0}+\beta_{1}DIF_{m,t}+\beta_{2}\;{\mathrm{Roa}}_{i,t}+\beta_{3}{\mathrm{Lev}}_{i,t}+\beta_{4}{\mathrm{Size}}_{i,t}+\beta_{5}\;{\mathrm{Growth}}_{i,t}\\ \;+\beta_{6}PPE_{i,t}+\beta_{7}\;{\mathrm{Age}}_{i,t}+\beta_{8}\;{\mathrm{Joint}}_{i,t}+\beta_{9}\;{\mathrm{Mshare}}_{i,t}\\ \;+\beta_{10}\;\mathrm{Inde}p_{i,t}+\beta_{11}PGDP_{m,t}+d_{j}+u_{i}+\delta_{i,t} \end{array}$$ (2)

$$\begin{array}{l} {\mathrm{Innovation}}_{i,t}=\gamma_{0}+\gamma_{1}DIF_{m,t}+\gamma_{2}SA_{i,t}+\gamma_{3}\;{\mathrm{Roa}}_{i,t}+\gamma_{4}\;{\mathrm{Lev}}_{i,t}+\gamma_{5}{\mathrm{Size}}_{i,t}\\ \;+\gamma_{6}\;{\mathrm{Growth}}_{i,t}+\gamma_{7}PPE_{i,t}+\gamma_{8}Age_{i,t}+\gamma_{9}{\mathrm{Joint}}_{i,t}\\ \;+\gamma_{10}\;{\mathrm{Mshare}}_{i,t}+\gamma_{11}\;{\mathrm{Indep}}_{i,t}+\gamma_{12}PGDP_{m,t}+d_{j}+u_{i}+\eta_{i,t} \end{array}$$ (3)

The variables and indicators in models (2) and (3) are described above. According to the mediating effect model principle, the total, direct, and indirect effects *β*_1_*γ*_2_ should satisfy the following equation: *α*_1_ = *γ*_1_+*β*_1_*γ*_2_. Combined with the theoretical analysis and hypotheses in the previous paper, the regression coefficient *γ*_2_ is expected to be negative, indicating that reducing the level of corporate financing constraints can promote SMEs’ technological innovation; financing constraints likely play a part in the mediating effect, as the absolute value of *γ*_1_ is smaller than the total value of *α*_1_.

To verify the reliability of the findings, based on the aforementioned empirical regressions, this paper tested the robustness of the role of digital finance in influencing corporate innovation. As the growth of digital finance relies mainly on the innovation of new generation digital technology, it is essentially driven by the technological innovation of enterprises. Therefore, companies with strong innovation capability may in turn enhance the development of digital finance, thus giving rise to the endogeneity problem emerging from reverse causality. In this paper, the explanatory and control variables were transformed with a one-period lag to eliminate the interference of reverse causality on the empirical results. The regression Model (4) was set as follows.


$$\begin{array}{l} {\mathrm{Innovation}}_{i,t}=\alpha_{0}+\alpha_{1}DIF_{\cdot }L_{m,t}+\alpha_{2}\;{\mathrm{Roa}}_{\cdot }L_{i,t}+\alpha_{3}\;{\mathrm{Lev}}_{\cdot }L_{i,t}+\alpha_{4}\;{\mathrm{Size}}_{\cdot }L_{i,t}+\alpha_{5}\;{\mathrm{Growth}}_{\cdot }L_{i,t}\\ +\alpha_{6}PPE_{\cdot }L_{i,t}+\alpha_{7}Age_{\cdot }L_{i,t}+\alpha_{8}J{\mathrm{oint}}_{\cdot }L_{i,t}+\alpha_{9}M\;{\mathrm{share}}_{\cdot }L_{i,t}\\ +\alpha_{10}\;{\mathrm{Indep}}_{\cdot }L_{i,t}+\alpha_{11}PGDP_{\cdot }L_{m,t}+d_{j}+u_{i}+\varepsilon_{i,t} \end{array}$$

## IV. Results and discussion

### 1. Empirical results on the impact of digital finance on SMEs’ innovation

Table 2 presents the outcomes of the benchmark empirical regression of the impact of digital finance on SMEs’ innovation. As seen in Column (1) of the table, the level of digital finance development has a significantly positive effect on SMEs’ R&D innovation incentives at the 1% level. One possible explanation is that digital finance, by combining digital technology with financial services and applying a new third-party credit information assessment system, has helped SMEs and banks to reduce the information asymmetry between them, compensate for the lack of information (such as credit approval) faced by SMEs in their previous lending operations, and improve the demand for funds needed by enterprises to carry out innovative activities.

**Table 2:** The test results of baseline regression and the mediating effect.

	(1)	(2)	(3)
	Innovation	SA	Innovation
DIF	0.0262***	-0.00268***	0.0277***
	(-3.68)	(-3.14)	(-3.41)
SA			-4.3237**
			(-2.20)
ROA	-0.1768***	0.0056***	-0.174***
	(-11.45)	(-6.52)	(-11.45)
Lev	-0.0949***	-0.0056*	-0.09859***
	(-16.56)	(-1.85)	(-16.26)
Size	-0.1952	0.3152***	1.1652*
	(-1.615)	(-308.52)	(-1.85)
Growth	0.0052**	-0.0000***	0.0012**
	(-2.56)	(-3.36)	(-2.52)
PPE	-0.0756***	0.0062**	-0.0752***
	(-9.44)	(-2.60)	(-2.935)
Age	-0.0226	-0.0326***	-0.1952**
	(-1.06)	(-237.90)	(-2.40)
Joint	0.3097*	0.302**	0.3033*
	-1.929	-1.2785	-1.8885
Mshare	0.00263	0.0001***	0.0049
	-0.709	-3.0826	-0.816
Indep	0.0243	0.0015	0.0248
	-1.559	(-0.00)	-1.5685
PGDP	-0.806*	-0.016***	-0.8587*
	(-1.68)	(-2.96)	(-1.79)
Constant	17.424***	16.2978***	-9.874
	-3.46	(-149.41)	(-0.73)
Year	controlled	controlled	controlled
Industry	controlled	controlled	controlled
AdjR^2^-squared	0.2852	0.985	0.2856

In addition, the coefficient of gearing (Lev) is significantly negative at the 1% level, indicating that firms operating with high leverage tend to have insufficient funds, and that the entrepreneurial and innovative activities of SMEs are limited, which is broadly consistent with the expected outcomes of the theoretical analysis. Growth is positively correlated at the 5% level of significance, and the coefficients of Size and Age are both negative in sign, suggesting that young firms with high growth tend to innovate more. The ratio of firm-fixed assets to firm innovation is significantly negative at the 1% level, implying that firm innovation does not depend on higher fixed assets.

### 2. Testing the mediating effect of financing constraints

According to the process of testing the mediating effect, first, the total impact of digital finance on SMEs’ innovation was tested. The results are shown in Column (1) in Table 2. Second, the empirical regression of Model (2) was conducted to test the correlation between digital finance and SMEs’ financing constraints. The results are displayed in Column (2) in Table 2 above, which demonstrates that the degree of digital finance development is significantly and negatively correlated with the financing constraints faced by companies at the 1% significance level. One possible explanation for this phenomenon is that digital financial services provide more choices for enterprise financing, and SMEs can choose the financing method that best meets their needs according to their actual business requirements, which helps companies to solve the financing constraint problem to a certain extent and finally achieve a more efficient allocation of financial resources. Finally, the results of Model (3) are displayed in Column (3) of Table 2 above. The findings show that financing constraints do play a partially mediating role, thus explaining the transmission mechanism of digital financial development to promote SMEs’ innovation and verifying Hypothesis 1.

### 3. Heterogeneity analysis of the nature of property rights and geographic distribution

According to the theoretical analysis, digital finance will promote enterprise innovation to different degrees depending on a company’s property rights. On the one hand, this is because, under the existing political and economic system, state-owned enterprises are more inclined to be provided with credit support by banks and the government due to their unique characteristics. On the other hand, with the improvement of the economic environment, private companies are better able to achieve a rational allocation of financial resources and have higher optimization efficiency than state-owned ones.

The empirical regression results are portrayed in columns (4) and (5) of Table 3, which show that digital finance significantly promotes technological innovation at the 1% level for private enterprises. Nevertheless, this finding does not hold for state-owned companies, as digital finance significantly inhibits technological innovation at the 1% level. Further, this finding does not hold for SOEs, as digital finance significantly inhibits their technological innovation at the 1% level. The reason is that the emergence of digital finance and innovative financial models has made up for the shortcomings of the traditional financial system in serving SMEs, and has contributed significantly to diversifying China’s financial market. Chinese state-owned enterprises are inevitably slower to transform than private ones due to their inflexible management compared to private companies and their size, so when the economic environment is significantly improved, private enterprises can quickly seize the opportunities offered by digital finance, and access more external financing to accelerate their development and investment in R&D and innovation.

**Table 3:** Regression results of companies’ property rights and geographic heterogeneity.

	(4)	(5)	(6)	(7)	(8)
	Private	State-owned	East	Middle	West
DIF	0.0265***	-0.0194**	0.0163***	0.0002**	0.00015**
	(2.65)	(2.25)	(2.34)	(2.05)	(2.86)
ROA	-0.1652***	-0.2358***	-0.6523***	-0.1456***	-0.2434***
	(-9.88)	(-3.25)	(-9.64)	(-4.15)	(-3.68)
Year	controlled	controlled	controlled	controlled	controlled
Industry	controlled	controlled	controlled	controlled	controlled
AdjR^2^-squared	0.265	0.2896	0.297	0.249	0.235

There is a significant concentration of financial resources in China due to the large differences in regional institutional growth. To sincerely portray the heterogeneity of the development of digital finance in driving innovation among enterprises across regions, this paper divided the original sample into three subsamples: east, central, and west, and the regression outcomes of the three subsamples are presented in Columns (6), (7), and (8) in Table 3. The empirical results reveal geographic heterogeneity in the impact of digital finance on Chinese SMEs’ innovation, with the incentive effect of digital finance on SMEs’ innovation in the eastern region being significantly positive at the 5% level. In contrast, for SMEs in the central and western areas, the DIF coefficient is much smaller and less significant than in the eastern region, indicating that the development of digital finance in the western and central parts has had little effect because the digital infrastructure and resource factor endowment of the western and significant regions are not as substantial as those of the eastern areas.

### 4. Robustness test

#### 4.1 One-period lag

To verify the reliability of the findings, based on the aforementioned empirical regressions, this paper tested the robustness of the role of digital finance in influencing corporate innovation. Since the development of digital finance mainly relies on the innovation of new generation digital technology—which essentially depends on promoting companies’ technological innovation—enterprises with strong innovation capability may in turn improve the development of digital finance, thus giving rise to the endogeneity problem brought about by reverse causality. In this paper, the explanatory and control variables were lagged one-period transformed to eliminate the interference of reverse causality in the empirical results.

The regression results after the one-period lag treatment are depicted in Table 4. The level of digital finance development in the lagged period still promotes SME innovation at the 5% significance level, implying that the previous findings still hold after endogenous interference is eliminated.

**Table 4:** The regression results of the endogeneity problem.

	(9)
	Innovation
L.DIF	0.0164**
	(-2.85)
L.ROA	-0.1289**
	(0.59)
L.Lev	-0.1048***
	(-15.02)
L.Size	-0.1485
	(-0.25)
L.Growth	0.0006
	(-0.48)
L.PPE	-0.0356***
	(-9.38)
L.Age	-0.0048
	(-0.25)
L.Joint	0.2874
	(-1.55)
L.Mshare	0.0001
	(-0.01)
L.Indep	0.0148
	(-1.02)
L.PGDP	-0.7085
	(-1.56)
Constant	14.565***
	(3.94)
Year	controlled
Industry	controlled
AdjR^2^-squared	0.268

#### 4.2 The replacement of the variables

First, the core explanatory variables were replaced. Then, the subvariable breadth of coverage (Breadth) of the digital finance index reflected each region’s perfection and range of digital infrastructure construction. Since digital finance development eliminates the constraints of time and space, breadth of coverage can, to a great extent, approximate the level of regional digital finance development, and it is reasonable to use the scope of coverage for substitution. The regression results, in Column (10) of Table 5 below, reveal that the range of digital finance coverage has a significant promotion effect on enterprise innovation, which is consistent with the previous results.

**Table 5:** The regression results of the endogeneity problem.

	(10)	(11)	(12)	(13)
	Innovation	Innovation	Innovation	Innovation
DIF		0.056**	0.085**	0.048***
		(2.61)	(2.65)	(2.52)
Breadth	0.052***			
	(5.57)			
Year	controlled	controlled	controlled	controlled
Industry	controlled	controlled	controlled	controlled
AdjR^2^-squared	0.264	0.256	0.214	0.252

Second, the explanatory variables were replaced. Since R&D innovation activities are highly uncertain, there is a risk of overestimation when measuring them directly from the perspective of R&D investment. Hence, to eliminate the possibility of overestimating innovation capability, this paper used the natural logarithm of the total number of patent applications in the current period (Patent) instead of existing indicators based on the current literature to further test the robustness of the impact of digital finance on corporate innovation. The regression results, in Column (11) of Table 5, demonstrate that the regression coefficient of digital finance regarding the number of patent applications is significantly positive, which is consistent with the regression results from the perspective of innovation input in the previous paper.

Finally, control variables were added. Foreign direct investment (FDI) affects companies’ access to external financing to a certain extent. Foreign capital can effectively solve this problem when SMEs face capital needs and cannot obtain support from the banking system. At the same time, the inflow of advanced alien technology and foreign capital jointly promotes enterprise innovation. Based on this, this paper regressed the data of FDI at each provincial level as a new control variable. The regression results are displayed in Column (12) of Table 5 below. The regression coefficients of both the level of digital finance development and FDI are significantly positive, indicating that both can drive corporate innovation, which is consistent with the previous paper.

#### 4.3 Converting the balance panel

Given that the sample data are missing or discontinuous in some years, this paper converted the unbalanced panel data into balanced panel data. As a result, it obtained a sample of 2,335 flat panels with five-year continuity from 2011 to 2020. The regression results, in Table 5 of Column (13), reveal that the previous conclusion still holds after converting the sample into a balanced panel, meaning that digital finance can significantly promote firm innovation. The above proves the robustness of the findings.

#### 4.4 Instrumental variables approach

In the robustness test approach, described above, the core explanatory variables were treated with a lag of one period to reduce the effect of endogeneity due to reverse causality. However, endogeneity bias can be caused by omitted variables in the empirical process, in addition to reverse causality. Therefore, this paper drew on Hsu et al. (2014), used provincial internet penetration (Hlw) as the instrumental variable, applied two-stage east squares regression, and re-estimated the model using two-stage least squares regression.

Before performing the two-stage least squares estimation, first, the Hausman test was applied to determine whether digital finance was an endogenous explanatory variable. The test results are shown in Table 6, where the Hausman statistics in Model X (2) are all significant at the 10% level, indicating that the variable of digital finance is an endogenous explanatory variable. Second, the LM test was used to test for non-identifiability; the LM statistics are all significant, thereby rejecting the non-identifiability hypothesis. Finally, the F test was applied to determine the validity of the selection of instrumental variables. The main variables in Model X (1) are significant at the 1% level. The F-statistic is 194.56, implying that the instrumental variable of internet penetration is strongly correlated with the endogenous explanatory variable of digital finance. The second-stage regression results reveal that the regression coefficient for digital finance in Model X (2) is significantly positive at the 5% level. Hence, the finding that digital finance drives technological innovation in SMEs is significant, whether grounded in the baseline or instrumental variables regression.

**Table 6:** Endogeneity treatment: Two-stage least squares instrumental variables method.

Variables	Estimation of instrumental variables: the first stage	Analysis of instrumental variables: phase 2
	DIF	Innovation
DIF	-	0.749**
	-	(2.19)
Hlw	0.118***	-
	(4.08)	-
Year	YES	YES
R^2^	0.528	0.586
F	194.56***	-
Hausman	-	5.815***
LM	-	16.418**

## V. Conclusions and recommendations

### 1. Conclusion

This paper explored and tested the mechanism of the degree of digital finance development affecting the R&D innovation of SMEs from multiple perspectives using the microdata of Chinese GEM-listed companies from 2011 to 2020. Moreover, the paper examined the impact path between the two based on financing constraints. The findings are as follows.

First, digital finance plays a significantly favorable influence in helping SMEs innovate and stimulate innovation. The higher the level of digital finance development, the more active corporate innovation is. Second, financing constraints play a partially mediating effect in promoting SMEs’ innovation. Digital finance mainly relies on advanced digital technology to tap various “soft” information for credit approval, helping to reduce information asymmetry between financiers, thus alleviating the financing constraints on SMEs and ultimately stimulating their innovation dynamics. Third, this paper scrutinized the heterogeneity of digital finance affecting SMEs’ innovation from the perspectives of property rights and geographic distribution. The results suggest that the development of digital finance has a more significant incentive effect on private companies’ innovation. For enterprises in different regions, the digital finance product in the eastern part also has a more substantial incentive effect on enterprise innovation due to the advantage of its economic system.

### 2. Recommendations

This paper proved that digital finance development indirectly helps SMEs’ innovation activities by assisting them in solving financing problems, which provides new inspiration for SMEs to alleviate financing constraints and stimulate innovation. This paper includes the following four policy recommendations rooted in the above results.

First, in the face of the switch between old and new dynamics, the shortcomings and contradictions of the traditional financial system in serving the natural and innovative economy have become more prominent, and the innovation drive has become more critical than ever. In this context, the Chinese government should introduce more industrial policies to encourage the development of digital technology, accelerate the industrial integration of digital technology and finance, make up for the shortcomings of traditional financial services, and build a more comprehensive, diversified, and inclusive financial services ecology. The government should introduce more policies to guide the empowerment of digital technology toward finance; broaden the depth, breadth, and precision of financial services; realize the quality and efficiency of financial services; and better support the development of China’s real economy and innovation economy. Second, as the main body of innovation activities in the market, private companies have more robust innovation vitality than state-owned enterprises. Thus, the government should formulate and launch policies related to encouraging innovation in a targeted manner and strengthen the protection of intellectual property rights. Third, due to regional clustering in China’s finances, companies’ innovation vitality in the central and western regions is insufficient.

On the one hand, China should actively promote the construction of a new generation of digital infrastructure in the central and western parts of the country to improve its coverage rate and lay the foundation for the long-term growth of digital finance. On the other hand, China should accelerate the improvement of economic system construction and the economic environment in the central and western parts of the country, guide financial institutions to eliminate the discrimination of financing for innovative subjects, and provide institutional guarantees for the reasonable allocation of financial resources. Fourth, digital finance is a new financial services innovation model, and its development in the real economy and innovation economy to promote its role is worthy of recognition to prevent the possible financial risks brought about by hidden dangers. Regulators can use digital technology to improve regulatory policies and identify, prevent, and control risks so that digital finance can develop in an orderly, healthy manner under the regulatory framework.

## Supporting information

S1 Data (XLSX)
